# Multiple Levels of Letter Representation in Written Spelling: Evidence From a Single Case of Dysgraphia with Multiple Deficits

**DOI:** 10.1155/2005/741623

**Published:** 2006-01-09

**Authors:** Marie-Pierre de Partz, Aliette Lochy, Agnesa Pillon

**Affiliations:** ^1^Université Catholique de LouvainBelgium; ^2^Centre de Revalidation Neuropsychologique des Cliniques Universitaires Saint-LucBruxellesBelgium; ^3^Fonds National de la Recherche ScientifiqueBelgium; ^4^F.C. Donders Centre for Cognitive NeuroimagingNijmegenthe Netherlands

**Keywords:** Acquired dysgraphia, graphemic buffer, letter substitution errors, grapheme, letter form, letter stroke

## Abstract

In this paper, we report a detailed analysis of the impaired performance of a dysgraphic individual, AD, who produced similar rates of letter-level errors in written spelling, oral spelling, and typing. We found that the distribution of various letter error types displayed a distinct pattern in written spelling on the one hand and in oral spelling and typing on the other. In particular, noncontextual letter substitution errors (i.e., errors in which the erroneous letter that replaces the target letter does not occur elsewhere within the word) were virtually absent in oral spelling and typing and mainly found in written spelling. In contrast, letter deletion errors and multiple-letter errors were typically found in oral spelling and very exceptional in written spelling. Only contextual letter substitution errors (i.e., errors in which the erroneous letter that replaces the target letter is identical to a letter occurring earlier or later in the word) were found in similar proportions in the three tasks. We argue that these contrasting patterns of letter error distribution result from damage to two distinct levels of letter representation and processing within the spelling system, namely, the amodal graphemic representation held in the graphemic buffer and the letter form representation computed by subsequent writing-specific processes. Then, we examined the relationship between error and target in the letter substitution errors produced in written and oral spelling and found evidence that distinct types of letter representation are processed at each of the hypothetized levels of damage: symbolic letter representation at the graphemic level and representation of the component graphic strokes at the letter form processing level.

## 1. Introduction

What kinds of letter representation are involved in the process of written spelling? That is, what kinds of letter information have to be processed in order to convert the knowledge of how a word has to be spelled into actual lines and curves drawn on a piece of paper? At least two distinct levels of letter representation and processing are assumed within current cognitive models of the writing system [19,25,30,41]: first, amodal (i.e., modality-independent) graphemic representations, subserving both written and oral spelling, and including information about the identity and position of the graphemes within the word; second, abstract (i.e., effector-independent) representations of letter form, involved in written spelling only, and specifying information required to assign a specific form to graphemes. Here we report the case study of a dysgraphic individual, AD, whose pattern of performance and errors in written and oral spelling can be described as resulting from damage to each of these letter representation levels, thus providing additional support for

ISSN 0953-4180/05/$17.00 © 2005 – IOS Press and the authors. All rights reserved the representational distinction assumed within these models.

The assumption that the writing system includes multiple and distinct representations of letter information, is drawn from detailed analyses of the impaired spelling performance of dysgraphic subjects. With reference to a hypothetical functional architecture of the spelling process, such as the one depicted in Fig. 1, these analyses first aimed at localizing the deficit of the subjects to one or more components within the architecture. This was achieved by contrasting the subjects' performance in several spelling tasks (e.g., copy, writing on dictation, oral spelling, etc), each task being supposed to recruit a distinct set of functional components. Then, the characteristics of the subjects' errors were used to draw inferences about the kind of representations that are processed at the level of the affected component (see [9,45] for arguments in favour of this methodological paradigm that uses data from impaired performance to constrain normal processing theories).

The hypothetical functional model depicted in Fig. 1 – which also informs the single-case study presented here – first distinguishes between the representations and processes involved in spelling familiar words and those used to generate plausible spellings for unfamiliar items. Thus, the spelling of familiar words may be retrieved by first accessing their meaning and then their corresponding lexical orthographic form stored in the orthographic output lexicon. Plausible spellings for unfamiliar items (for which there is no stored orthographic representation) can be generated by relying on knowledge of phoneme-grapheme correspondences and applying sublexical phonology-to-orthography conversion (POC) procedures. Representations resulting from either lexical or sublexical POC processing are assumed to consist of a sequence of amodal graphemes that are held in a temporary memory system – the graphemic buffer – while their corresponding letter forms or names are being processed by the subsequent, modality-specific, mechanisms dedicated to either written or oral spelling [8,19,30,31,39]. Thus, in written spelling, the amodal graphemes are assigned a specific form (case, font, and shape), which are then written down through execution of neuromuscular commands. In oral spelling, the amodal graphemes are converted into the phonological representations corresponding to their letter names, which in turn activate articulatory programs to speak them aloud [31]. Let us add that, although little is known in relation to typing, modality-specific mechanisms dedicated to this modality of spelling output may be assumed, which would specify, for example, information about the letter location on the keyboard together with, possibly, the appropriate digit to reach it.

Neuropsychological evidence supporting the existence of an amodal level of letter representation and processing is provided by numerous reports of patients producing similar rates and kinds of errors in both oral and written spelling, for both words and nonwords (e.g. [2,5–7,11,13,17,26–28,32,34,35,37–39,43, 44,46]). These patients' spelling errors in both modalities of spelling and for both words and nonwords were analysable as the consequence of incorrect selection or ordering of individual letters, i.e., letter substitutions (e.g., STU**D**Y → STU**T**Y), deletions (e.g., DISCOVERY → DICOVERY), additions (e.g., PORCELAIN → PORCE**R**LAIN), and transpositions (e.g., NIG**HT** → NIG**TH**). Moreover, in most of these case reports, spelling accuracy was not affected by lexical factors like word frequency, age of acquisition, imageability, and orthographic neighbourhood size, which suggested that the impairment was located at a representation and processing level that follows access to the orthographic representations stored in the lexicon (but see, for discussion [6,43]). This amodal letter representation and processing level was further conceived of as a temporary memory system – a graphemic buffer – on the basis of two characteristic features of the patients' spelling performance: spelling errors occurred with increased frequency as a function of the stimulus length and did not distribute evenly across the various letter positions in the stimulus. In some patients, the errors were mostly located at the end of the words, which could reflect a left-to-right read-out process operating over a too rapidly decaying memory trace [27,44]. In other cases, the errors mostly involved letters in the middle of the words [7], which was accounted for by the letters in internal positions being subjected to interference from more neighbour letters than the letters located at extreme positions [52].

Further to these patients whose pattern of spelling performance suggested damage at an amodal level of processing, patients have been reported with damage likely due to modality-specific mechanisms dedicated to either written or oral spelling: some patients were unable to retrieve the name of the graphemes present in a word or nonword, while being perfectly able to write down the same items (e.g. [10]); others were impaired in written spelling, while their performance in oral spelling was relatively spared (e.g. [1,3,4,15,18, 22–24,29,36,40,42,49,53]).

As regards the specific mechanisms involved in written spelling, Ellis [19] and Margolin [30] put forward

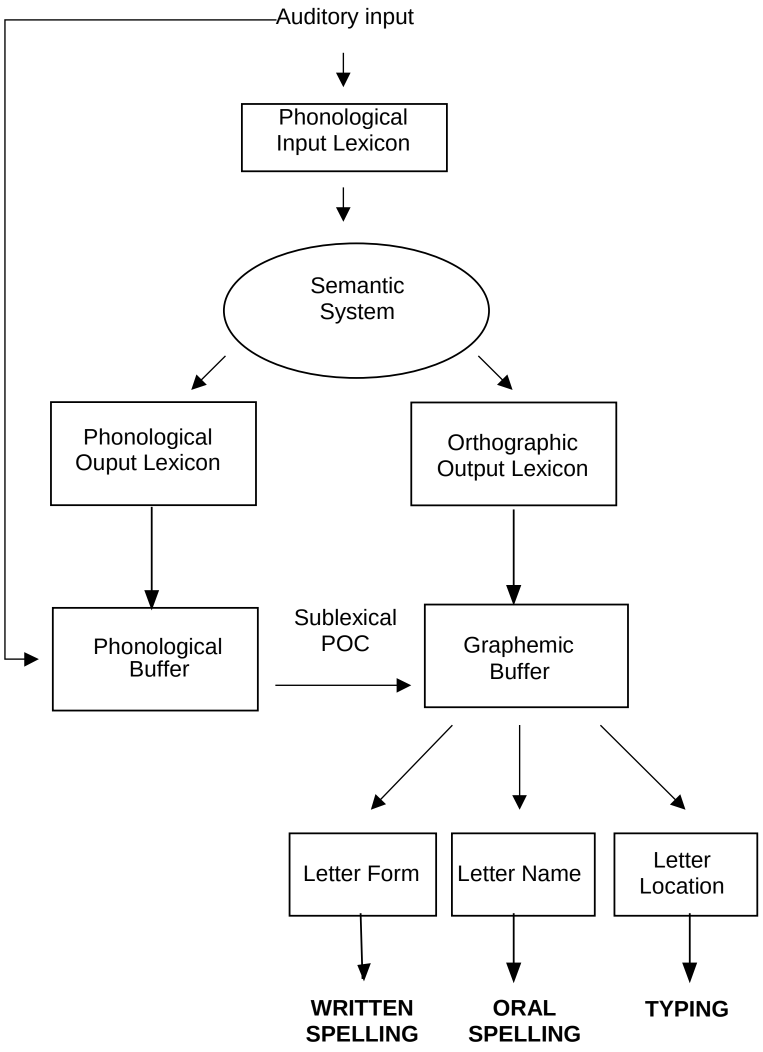

Fig. 1. Schematic depiction of the cognitive architecture of the spelling system (POC = Phonology-to-orthography conversion procedures).

the hypothesis that amodal graphemes are assigned form by two distinct and successive processes (Fig. 2). First, letter *shapes* are retrieved from a long-term "allographic store" representing letter shapes in terms of their visuospatial features. At that level, a specific shape among the various variants or allographs of a given grapheme is selected, for example, the lowercase "a" or uppercase "A", or the corresponding cursive or print shape. It is at the subsequent level, the "graphic motor pattern" level, that the *sequence of strokes* required to write down the selected allograph is retrieved. Letter information, at this level, thus includes the direction, relative size, position, and order of strokes required to give a written form to a given allograph. The authors motivated this distinction between two letter-form representation levels, one specifying visual shape and the other the sequence of graphic strokes, by cases of patients whose contrasting patterns of writing errors suggested damage to one or the other of these levels. Patients who were unable to have a mental image of the shape of letters and made, in written spelling, case mixing errors or letter substitution errors between letters having a similar shape [15,23,24,36,48,49] would have damage at the level of the allographic store. On the other hand, patients who had no difficulty in selecting the appropriate case but made numerous well-formed letter substitution errors in written spelling only (e.g. [18,20,22,29,48]) or wrote forms that were not real letters [3,53] would have a deficit in retrieving the appropriate sequence of letter strokes (but see [40] for a discussion and an alternative account for such patterns).

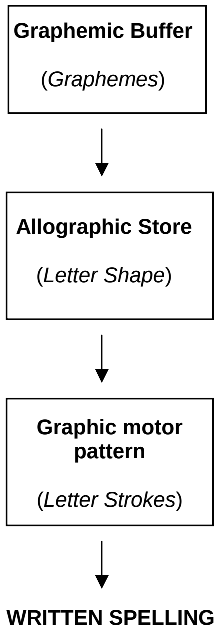

Fig. 2. Post-lexical components involved in written spelling, according to Ellis [19] and Margolin [30].

Thus, each of the three letter processing levels that have been hypothetized to date are assumed to represent and process a specific *content* and *type* of letter information: the level of the graphemic buffer represents letter identity (and position within the word) as amodal, symbolic representations, the level of the allographic store represents letter shape as visuospatial features, and the subsequent level, letter form as a sequence of strokes required to write down a specific letter form.

Rapp and Caramazza [40] evaluated these assumptions by contrasting the spelling error patterns of two dysgraphic subjects with damage at the level of the graphemic buffer, with that of two dysgraphic subjects with damage at a postbuffer level. Focusing on the uppercase letter substitution errors made by these patients in written spelling, they scored each of the target-error letter pair as being either visuospatially similar (e.g., D → C), similar in terms of the features of their component strokes (e.g., L → T), ambiguous (similar according to both visuospatial and stroke features; e.g., F → T), or unrelated (no visuospatial nor stroke feature similarity; e.g., B → C). The visuospatial similarity metric was based on a confusion matrix obtained by asking unimpaired individuals to identify briefly displayed uppercase letters and the stroke similarity metric was based on an analysis of the actual strokes produced by the patients for each uppercase letter. Results indicated that letter substitution errors produced by the patients with damage to the graphemic buffer displayed no physical similarity to the intended target, whether in terms of visuospatial or component stroke features. On the other hand, erroneous letters produced by the patients with a postbuffer deficit were physically similar to the target, and similarity was based on features of the component strokes of the letters, not their visuospatial features. This study thus provides support for the distinction between a symbolic representation of letters at the graphemic buffer level and a representation of letter form in terms of component graphic strokes at a modality-specific level involved in written spelling. Furthermore, although this study does not rule out the existence of additional, visuospatial representations of letter form at the level of a allographic store, for example, it nevertheless points to the type of evidence that is needed to support this hypothesis. Since a significant number of letter pairs that are visuospatially similar are also similar in terms of their component strokes, analyses of letter substitution errors made by patients having damage to the visuospatial "allographic store" (e.g. [20, 48,49]) should be able to show that the "shape similarity" observed between targets and errors is based on visuospatial features, not on stroke features – evidence that is still lacking to date.

Inspired by this work by Rapp and Caramazza, we examined, in the case study reported below, the pattern of lowercase letter substitution errors produced by a single patient in written and oral spelling and found further evidence in support of the distinction between symbolic letter representations and letter form representations specifying the component strokes required to produce the written form. Although the present patient had impaired performance in both written and oral spelling (and typing), the distribution of his letter-level errors – especially when, among them, letter substitution errors were split into contextual and noncontextual errors – differed across both spelling tasks. This pattern lead us to hypothesize two distinct loci of damage: the graphemic buffer, which impaired performance in both written and oral spelling (and typing), and the letter-form processing level, which further impaired performance in written spelling. We then show that in a specific type of letter errors found in written spelling only, namely, the noncontextual letter substitution errors, target-error letter pairs were similar in terms of their component strokes, whereas in the contextual letter substitution errors, found in written and oral spelling, target-error letter pairs bore no physical similarity. Thereby, we replicated the findings of Rapp and Caramazza in the context of a study carried out

Table 1
AD's scores to standard neuropsychological tests

| Neuropsychological test | AD's score | Controls' score Mean (SD) | AD's score (z-score or percentile, P) |
|---|---|---|---|
| **ATTENTION** | | | |
| *TEA* [54] | | | |
| Lateralised Reaction Time | | | |
| Total space RT | 292 ms | 307 ms (70) | 0.21 |
| Left RT | 296 ms | | |
| Right RT | 288 ms | | |
| Divided attention | | | |
| RT | 842 ms | | P. 5 |
| Errors | 7 | | P. 3 |
| *D2* | | | |
| Sustained attention | | | |
| Total Raw Score | 265 | | P. 11 |
| Errors | 6 (2.3%) | | P. 90 |
| Total score minus errors | 259 | | P. 18 |
| Fluctuation | 11 | | P. 50 – P. 75 |
| **MEMORY** | | | |
| Digit span – Forward | 6 | 6.27 (1.2) | − 0.22 |
| Digit span – Backward | 5 | 4.18 (1.2) | 0.68 |
| Spatial span (Corsi) | 5 | | P. 50 |
| Spatial span (Span Supra span test) [51] | 7 | 8.88 (1.1) | − 1.7 |
| Brown Petterson | | | |
| 0 sec | 100% | 98.9% (3.5) | 0.31 |
| 5 sec | 100% | 80.6% (15.3) | 1.26 |
| 10 sec | 94.5% | 70.6% (24.7) | 0.97 |
| 20 sec | 88.9% | 66.1% (17.5) | 1.30 |
| Buschke 16 items [47] | | | |
| Free recall | 33 | 28.9 (6.8) | 0.60 |
| Cued recall | 15 | 15.8 (5.1) | 0.16 |
| Recognition | 16 | 15.7 (0.5) | − 0.6 |
| Span Supra-span test | | | |
| Supra span | 8 | 8 (4.5) | 0 |
| **LANGUAGE** | | | |
| Picture Naming | 90/90 | (0–7) | Perfect |
| Verbal fluency | | | |
| Semantic (Animals) | 39 | 36 (3.4) | 0.88 |
| Letter (P) | 26 | 16.5 (3.1) | 3.22 |
| Word-to-picture matching [16] | 63/64 | 61.8 (1.3) | 0.92 |
| Synonym pointing (written version) | 118/120 | 116.2 (1.7) | 1.05 |
| Sentence-to-picture matching | | | |
| Reversible (active, passive, relative) | 12/12 | | |
| Non reversible (relative) | 4/4 | | |

with a single patient, whose spelling errors were analysed with a different method for estimating visuospatial and stroke-feature similarity between letter pairs, which were lowercase, instead of uppercase, letters.

## 2. Case report

AD, a right-handed French-speaking male with a University degree in literature, was born in 1932. He suffered an infarct in July 1999. Templates were used to identify the lesion size and localization from the available CT-scan images (see Fig. 3) [14]. The lesion was approximately $4 \times 5 \times 4$ cm and involved Brodmann area (BA) 39, the posterior part of BA 40, and the most inferior part of BA 7 on the left side. Another smaller lesion (about 1.5 cm) was observed in the left frontal region (BA 6, mostly subcortical). This infarct resulted in a Gerstmann syndrome. Signs of apraxia were also reported such as difficulties in using real objects (e.g., cutlery and tools), facial, constructional, and dressing apraxia. Furthermore, AD complained of difficulties in writing and reading. These difficulties appeared very mild, but the patient's spouse asserted that AD was previously a very competent reader and writer.

Table 2
Composition of the eight lists of stimuli presented to AD in written spelling, oral spelling, and typing

| N° List | Variable | Number of items | Composition |
|---|---|---|---|
| List 1 | Frequency | 112 words | 56 high-frequency words (fq > 1000 per million)<br>56 low-frequency words (fq < 100 per million)<br>Matched for length (number of letters) |
| List 2 | Imageability | 60 words | 30 high-imageability words<br>30 low-imageability words<br>Matched for length (number of letters and number of syllables), frequency, and regularity of spelling |
| List 3 | Spelling regularity | 100 words<br>(repeated three times in written spelling and twice in oral spelling) | − 20 regular words (Degree of Orthographic Ambiguity = 0)<br>− 40 orthographically ambiguous words<br>(one phoneme is not written in the most frequent way ; DOA = 1)<br>− 20 orthographically ambiguous words<br>(2 phonemes are not written in the most frequent way ; DOA = 2)<br>− 20 irregular words<br>(one phoneme is written in an exceptional way)<br>Matched for frequency and length (number of letters and number of syllables) |
| List 4 | Grammatical class | 120 words | 60 nouns – 60 verbs<br>Matched for frequency and concreteness |
| List 5 | Length | 146 words<br>(repeated twice in written spelling, oral spelling, and typing) | 73 short words (3–5 letters)<br>73 long words (10–12 letters)<br>Matched for frequency |
| List 6 | Other | 65 words | Very long words (13 to 15 letters) |
| List 7 | Syllabic structure | 210 words | 105 words with single consonants (CV)<br>105 words with consonant clusters (CCV – CCCV)<br>Matched for frequency and length (all eight-letter words) |
| List 8 | Lexicality | 105 nonwords | Matched for length and syllabic structure with the set of 105 consonant-cluster words of List 7 |

In July 2001, AD suffered a second transitory stroke that caused a transitory left hemiparesia without other neurological signs (the neuropsychological examination carried out at that moment showed no peculiarity in comparison with that carried out after the first infarct).

Our experimental investigation was conducted between October 1999 and December 2000, except that one stimulus list (List 7, see description below) was administrated to AD in December 2001. During this period, AD's cognitive status remained stable.

The results of the standard neuropsychological exams carried out in October 1999 (i.e., when our investigation began) are summarized in Table 1. Although previous signs of apraxia had largely resolved, occasional hesitations in right-left distinction and residual difficulties in mental calculation were still noted. Selective and sustained attention was normal but divided attention was impaired. There was no evidence of unilateral neglect. Phonological and visual short-term memory and working memory were normal. Verbal long-term memory (recognition, free and cued recall) and visuospatial long-term memory (Span and Supraspan test) were also normal.

AD's performance on tests of language was normal, except for reading and spelling. Spontaneous speech was fluent with normal intonation, articulation, and lexical production. Grammatical complexity and vocabulary were within normal limits for his age and education. Repetition of words and sentences was intact. Visual confrontation naming, synonym production and word fluency tasks were all performed perfectly. In spoken and written word-to-picture matching tasks and in a synonym pointing task with concrete and abstract (spoken and written) nouns and verbs, AD responded quickly and accurately. In an auditory sentence-to-picture matching test, he demonstrated normal comprehension of reversible active, passive, and relative sentences.

Evaluation of reading performance revealed that AD read aloud letters, isolated words, and pseudowords rapidly and perfectly. There were no effects of frequency, imageability, grammatical class, or spelling regularity on performance. However, in reading aloud text, he produced occasional substitutions of function words, visual, and exceptionally semantic errors. Nevertheless, he spontaneously corrected most errors. On some occasions, AD had to read again previous portions of the text in order to achieve understanding, especially when he read quickly. On the whole, in spite

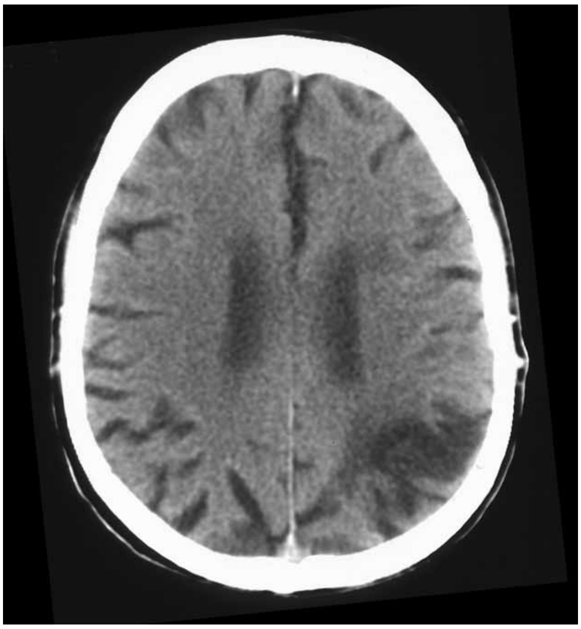

Fig. 3. CT-Scan image for AD.

of the very good reading performance demonstrated by AD in these tests, it was probably not as good as his premorbid reading skill.

AD's writing was fluid and without lexical, orthographic, or grammatical errors. However, the patient produced letter substitutions, deletions, additions, and transpositions in various spelling tasks: written spelling, oral spelling, written naming, and delayed copy. Given the similar letter error rates observed across the various modalities of spelling output, we at first hypothetized that AD's writing deficit was located at the level of the graphemic buffer. However, as we will show in the following section, a detailed investigation of his spelling uncovered a number of observations that were not fully consistent with this hypothesis, which we therefore qualified subsequently.

## 3. Experimental investigations

### *3.1. Analysis of AD's performance in written spelling, oral spelling, and typing*

#### *3.1.1. Materials and procedure*

AD's performance in written spelling, oral spelling, and typing was assessed with eight lists of stimuli. Each list was designed to evaluate the effect of a specific variable on his spelling performance (see Table 2). In total, AD was asked to write on dictation 1159 words and 105 nonwords. He was presented with 1059 words and 105 nonwords for oral spelling, and with 617 words for typing. Because AD had a peripheral auditory deficit, largely but not completely corrected by prosthesis, he was asked to repeat the stimuli that were dictated to him before writing them, in order to ensure he had correctly perceived them. In all the tasks, the patient was allowed to produce more than one response, that is, to self-correct a response if he wished to.

#### *3.1.2. Spelling accuracy*

*3.1.2.1. Scoring procedure*

When more than one response was produced, only the first one was scored as correct/incorrect. When the first response was an aborted response, it was always considered as an incorrect response, even when the patient then restarted and produced a full and correct response, and whether the fragment produced was correct (e.g., **CONTR**ÔLER (*to check*) → **CONTR** . . . CONTRÔLER) or not (e.g., **B**APTÊME (*baptism*) → **P** . . . BAPTÊME). However, just pausing within a word was not considered an aborted response and hence was scored as a correct response (e.g., CONTRÔLER (*to check*) → CONTR . . . ÔLER); in these cases, the patient stopped after having spelled a correct fragment and then went on spelling, but he did not restart from the beginning as in the cases of aborted responses.

*3.1.2.2. Results*

As indicated in Table 3, AD's spelling accuracy was similar in written spelling (89%), oral spelling (87%), and typing (85%). AD was generally aware of his errors. He self-corrected most written spelling and typing errors (respectively, 91% and 84% of errors were self-corrected), but he self-corrected less often in oral spelling (only 44% of the errors were self-corrected). After self-correcting, the percentage of AD's correct responses was 98% in written spelling, 91% in oral spelling, and 97% in typing.

Given the high rate of AD's correct responses, one question could be raised about whether his performance was impaired at all in comparison with control subjects. Therefore, a subset of 669 word stimuli from the various lists was presented in oral and written spelling to a control group composed of eight right-handed subjects (five men and three women), matched to the patient for age (mean = 65.8 years) and education level (all had a University degree in literature). These control subjects had a MMSE score equal or higher than 27/30 and performed within the normal range at the digit span

Table 3
Number (and percentages) of AD's correct responses in written and oral spelling and typing, in the various lists of stimuli contrasting word frequency, imageability, spelling regularity (DOA = Degree of Orthographic Ambiguity), length, grammatical class, syllabic structure, and lexicality

| List of stimuli (variable) | Written spelling | Oral spelling | Typing |
|---|---|---|---|
| List 1 (Word frequency) | | | |
| High Frequency | 51/56 (91) | 46/56 (82) | n.a. |
| Low Frequency | 46/56 (82) | 49/56 (87) | |
| List 2 (Imageability) | | | |
| High Imageability | 29/30 (97) | 21/30 (70) | 28/30 (93) |
| Low Imageability | 27/30 (90) | 25/30 (83) | 27/30 (90) |
| List 3 (Spelling regularity) | | | |
| DOA 0 | 57/60 (95) | 38/40 (95) | 31/40 (77) |
| DOA 1 | 113/120 (94) | 74/80 (92) | 70/80 (88) |
| DOA 2 | 55/60 (92) | 38/40 (95) | 37/40 (92) |
| Irregular | 46/60 (77) | 32/40 (80) | 29/40 (72) |
| List 4 (Grammatical class) | | | |
| Nouns | 54/60 (90) | 53/60 (88) | n.a. |
| Verbs | 50/60 (83) | 56/60 (92) | n.a. |
| List 5 (Length) | | | |
| 3–5 letters | 137/146 (94) | 141/146 (96) | 141/146 (96) |
| 10–12 letters | 120/146 (82) | 117/146 (80) | 111/146 (76) |
| List 6 | | | |
| 13–15 letters | 49/65 (75) | 41/65 (63) | 49/65 (75) |
| List 7 (Syllabic structure) | | | |
| CV | 94/105 (89) | 92/105 (88) | n.a. |
| CCV/CCCV | 100/105(95) | 95/105 (90) | |
| List 8 (Lexicality) | | | |
| Nonwords | 94/105 (92) | 96/105 (91) | n.a. |
| **TOTAL** | **1122/1264 (89)** | **1014/1164 (87)** | **523/617 (85)** |

and the Brown-Peterson tests. They were tested in the same conditions as AD, by alternating oral and written spelling but during two sessions of three hours. The scoring procedure was the same as that used for AD.

With this subset of words, AD scored 87% and 85% correct in written and oral spelling, respectively; the mean score of the control subjects was respectively 97% and 94%. A first set of analyses revealed that the control subjects performed significantly better in written than oral spelling ($t = 3.63$, $p < 0.01$, df $= 6$), whereas no significant difference was noted between both tasks in AD's performance ($\chi^2_{(1)} < 1$).[1] That the normal advantage of written over oral spelling was not found in the patient's performance might indicate that, in spite of his similar error rates in both tasks, he was more impaired, in fact, in written than oral spelling. The results of the data analyses performed with Crawford and Garthwaite's modified t-test [12] supported this conjecture: AD's performance was significantly impaired in comparison with the controls', both in written spelling (one-tailed $t = -4.42$, $p < 0.0001$, df $=$ 7) and oral spelling (one-tailed $t = -1.98$, $p < 0.05$, df $= 7$), and significantly more impaired in written than oral spelling (two-tailed $t = 4.06$, $p < 0.005$, df $= 7$).

### 3.1.3. *Effect of different variables on spelling accuracy*

In the three spelling tasks (see Table 3), AD's performance did not significantly vary as a function of lexicality (List 8), word frequency (List 1), imageability (List 2), or grammatical class (List 4) (all $\chi^2 < 1$). This pattern points to a post-lexical locus for the deficit. However, AD's performance in written spelling was significantly better with regular than irregular words (List 3; $\chi^2_{(1)} = 6.85$; $p < 0.01$), which is unexpected within this context. This effect is probably not, however, a genuine regularity effect. All but two errors AD produced with irregular words were in fact letter substitution errors, exactly like the errors he produced with regular words. Thus, for example, in the word CHORALE (*choir*), the irregularity in phonology-to-orthography mapping lies in the first two letters "CH", which are an

[1] When appropriate, the correction of Yates was applied in all $\chi^2$ reported in the paper.

exceptional mapping for /k/, usually wrote "C" or "K". The error AD committed in writing this word, namely, CHO**R**ALE (*choir*) → CHO**L** . . ., did not consist in a regularization error (e.g., **CH**ORALE → CORALE or **K**ORALE), but in a substitution affecting another letter in the word. In fact, as we will see, control subjects produced more regularization errors than AD.

More importantly, it appeared from the results obtained for List 5 that stimulus length affected AD's spelling accuracy in the three tasks. AD's performance was significantly better with short than long words in written spelling ($\chi^2_{(1)} = 8.31$; $p < 0.005$), oral spelling ($\chi^2_{(1)} = 17.71$; $p < 0.0001$), and typing ($\chi^2_{(1)} = 24.46$; $p < 0.0001$). However, when responses were scored as the number of correctly spelled letters over the total number of letters in the stimulus, the effect of stimulus length was no more significant in written spelling (98% correct letters in short words vs. 98% correct letters in long words; $\chi^2_{(1)} < 1$), while it was still significant in oral spelling (99% vs. 97%; $\chi^2_{(1)} = 4.80$; $p < 0.03$) and failed to reach significance in typing (99% vs. 98%; $\chi^2_{(1)} = 2.50$; $p = 0.11$).

The evidence so far points to a post-lexical locus for AD's deficit in spelling: AD was similarly impaired for words and nonwords, and lexical variables such as word frequency, imageability, and grammatical class did not affect his performance in the three spelling tasks. By reference to the functional architecture of the spelling system schematised in Fig. 1, the similar error rates observed in written spelling, oral spelling, and typing suggest the hypothesis that AD's impaired performance in these three tasks originated from damage to the graphemic buffer. However, there are a number of observations that make this hypothesis unsatisfactory. First, written spelling was relatively more impaired than oral spelling when compared to the control subjects' performance. Second, stimulus length did not similarly impact on AD's performance in the three spelling tasks. In particular, stimulus length had a highly significant effect on performance in oral spelling, which is consistent with the hypothesis that errors in that task are the result of damage to the graphemic buffer, but no significant effect in written spelling, which is inconsistent with the hypothesis that writing errors originated from the same locus of damage. These discrepant patterns suggest the possibility that AD's errors in written and oral spelling (and typing) in fact arose from a combination of distinct deficits. Two reasonable hypotheses might be entertained. The first is that AD's impaired performance in the three spelling tasks results from damage to all of the modality-specific mechanisms involved in written spelling, oral spelling, and typing. The second hypothesis is that AD's impaired performance results from damage to a common functional component involved in the three spelling tasks and impairing performance in these three tasks – the graphemic buffer – plus additional damage to a functional component specific to writing and further impairing performance in written spelling. A qualitative analysis of AD's errors, presented in the next section, will help us to choose between these two alternatives.

#### 3.1.4. Qualitative analyses of AD's errors

The error corpus submitted to the following analyses included all the erroneous responses produced by AD (that is, not only his first responses, as in the previous quantitative analyses). Thus, in total, there were 157 errors in written spelling, 146 errors in oral spelling, and 99 errors in typing. In the three tasks, most erroneous responses were word or nonword letter sequences that did not correspond to the pronunciation of the stimulus word (i.e., so-called phonologically implausible responses) and that could be described as letter-level errors.

##### 3.1.4.1. Distribution of the various error types

We distinguished three main kinds of errors: single-letter errors, multiple-letter errors and other errors (see examples of each error type in Table 4). Single-letter errors include letter substitutions, additions, deletions, and transpositions. Letter transposition errors concern misplaced letters, such as letter exchanges, in which two letters exchange their position, and letter shifts, in which one letter moves from one position to another. Importantly, among letter substitutions, we separated contextual from noncontextual errors:

- In contextual letter substitution errors, the letter that replaces the target letter occurs elsewhere in the stimulus, either later (anticipation errors, like, **T**APAGE (*noise*) → **P** . . .), earlier (perseveration errors, like VETE**R**INAIRE (*veterinarian*) → VÉTÉ**V**INAIRE), or both (ambiguous errors: CO**Q**UELICOT (*poppy*) → COCUELICOT).
- In noncontextual letter substitution errors, the letter that replaces the target letter does not occur elsewhere in the stimulus (TR**O**NC (*trunk*) → TR**A**NC).

Multiple-letter errors consist of responses in which more than one letter was erroneous. Other errors include (i) orthographic errors, namely, errors reflecting

Table 4
Examples of AD's spelling errors

| ERROR TYPE | EXAMPLES | |
|---|---|---|
| | TARGET | ERROR |
| **SINGLE-LETTER ERROR** | | |
| **SUBSTITUTION** | | |
| *Contextual* | | |
| Anticipation | TA**P**AGE (*row*) | **P** ... |
| Perseveration | VÉTÉ**R**INAIRE (*veterinarian*) | VÉTÉ**V**INAIRE |
| *Noncontextual* | | |
| Erroneous letter | TR**O**NC (*trunk*) | TR**A**NC |
| Non-letter | PA**TT**E (*leg*) | PA / / E |
| **ADDITION** | VICTUAILLE (*food*) | VI**N**CTUAILLE |
| **TRANSPOSITION** | | |
| Exchange | T**R**ANSPLANTATION (*transplantation*) | T**AR** ... |
| Shift | AUT**H**ENTIQUE (*authentic*) | AUTEN**H**TIQUE |
| **DELETION** | SYMP**T**ÔME (*symptom*) | SYMPÔME |
| **MULTIPLE-LETTER ERROR** | SALA**M**ANDRE (*salamander*) | _ALA**G** ... |
| **OTHERS** | | |
| ORTHOGRAPHIC | AGRA**F**EUSE (*stapler*) | AGRA**FF**EUSE |
| RESTART | **CONTR**ÔLER (*to check*) | **CONTR** ... CONTRÔLER |
| AMBIGUOUS | QUAR**A**NTE (*forty*) | QUAR**E**NTE |

lack (or loss) of knowledge about how a word is spelled (e.g., AGRA**F**EUSE (*stapler*) → AGRA**FF**EUSE, with AD commenting "I don't remember the correct spelling, one or two F"); (ii) restarts, namely, aborted responses in which the patient correctly spelled a portion of a stimulus, then stopped and spelled it again from the beginning without any error (e.g., **CONTR**ÔLER (*to check*) → **CONTR** . . . CONTRÔLER); and (iii) ambiguous errors, which could be classified in more than one error type (e.g., in QUAR**A**NTE (*forty*) → QUAR**E**NTE, the error could be classified either as a contextual letter substitution error or an orthographic error).

The results of the error analysis for word stimuli (Table 5) indicated that single-letter errors were the most frequent type of errors in the three spelling tasks. They amounted to 87%, 72%, and 79% of total errors in written spelling, oral spelling, and typing, respectively. More importantly, it appears that the error distribution differs between written and oral spelling (and typing). In written spelling, the majority of errors were contextual (37%) and noncontextual (29%) letter substitution errors; letter deletions were exceptional (6%). On the other hand, in oral spelling, the predominant types of errors were letter deletions (34%) and contextual letter substitutions (22%), noncontextual letter substitutions being very exceptional (1%). Moreover, in written spelling, a few instances (9%) of distorted letters were produced. These non-letters could be described as resulting from the omission of a stroke (5 instances of omission of the horizontal line in the letter "T"), addition of a stroke (4 instances, all at the beginning of the letter "Y"), substitution of a stroke (2 instances), or as distorted letters (3 instances, where the origin of the errors was unclear). Production of non-letters like these suggests that a post-graphemic, modality-specific level of letter processing was mildly impaired in AD.

The distribution of AD's errors for nonword stimuli was very similar to that observed for word stimuli. In written spelling, most errors were letter substitutions (8/11), in particular noncontextual letter substitutions (6/11) whereas, in oral spelling, most errors were letter deletions (8/11) and no one letter substitution was noted.

So far the data analyses for the written spelling task include responses in cursive lowercase script only. To have a more complete picture of AD's performance in written spelling, we asked him to write in uppercase the words from List 5 (twice) and List 3, which made a total of 392 words. The results are presented in Table 6. The patient produced 26% of errors when writing in uppercase. The distribution of these errors across the different error types was not significantly different from the error distribution observed for lowercase script ($\chi_{(3)} < 1$): the majority of errors were contextual (38%) and noncontextual (24%) letter substitutions, letter deletions being exceptional (7%).

Finally, we compared the distribution of AD's errors with that of the controls, for the subset of 669 word stimuli presented both to AD and the controls in written

Table 5
Distribution of AD's errors in written spelling, oral spelling, and typing, for word stimuli

| ERROR TYPE | Written spelling | | Oral spelling | | Typing | |
|---|---|---|---|---|---|---|
| | N | % | N | % | N | % |
| **SINGLE-LETTER ERROR** | | | | | | |
| **SUBSTITUTION** | **117** | **74** | **32** | **23** | **43** | **43** |
| **Contextual** | **58** | **37** | **32** | **22** | **38** | **38** |
| Anticipation | 50 | 32 | 22 | 15 | 25 | 25 |
| Perseveration | 5 | 3 | 3 | 2 | 4 | 4 |
| Anticipation or perseveration | 3 | 2 | 7 | 5 | 9 | 9 |
| **Noncontextual** | **45** | **29** | **2** | **1** | **5** | **5** |
| **By a non-letter** | **14** | **9** | – | – | – | – |
| **ADDITION** | **7** | **4** | **14** | **10** | **14** | **14** |
| **TRANSPOSITION** | **4** | **3** | **8** | **5** | **8** | **8** |
| Exchange | 4 | 3 | 6 | 4 | 8 | 8 |
| Shift | – | – | 2 | 1 | – | – |
| **DELETION** | **9** | **6** | **49** | **34** | **13** | **13** |
| **MULTIPLE-LETTER ERROR** | **6** | **4** | **28** | **19** | **15** | **15** |
| **OTHERS** | **14** | **9** | **13** | **9** | **6** | **6** |
| ORTHOGRAPHIC | 6 | 4 | 5 | 3 | 3 | 3 |
| RESTART | 7 | 4 | 6 | 4 | – | – |
| AMBIGUOUS | 1 | 1 | 2 | 1 | 3 | 3 |
| **TOTAL ERRORS** | **157** | **100** | **146** | **100** | **99** | **100** |

Table 6
Distribution of AD's errors in writing in lowercase and uppercase script

| ERROR TYPE | Lowercase script | | Uppercase script | |
|---|---|---|---|---|
| | N | % | N | % |
| **SINGLE-LETTER ERROR** | | | | |
| **SUBSTITUTION** | **117** | *74* | **76** | *74* |
| **Contextual** | **58** | *37* | **39** | *38* |
| *Anticipation* | 50 | *32* | 37 | *36* |
| *Perseveration* | 5 | *3* | 2 | *2* |
| *Anticipation and/or perseveration* | 3 | 2 | – | – |
| **Noncontextual** | **45** | *29* | **25** | *24* |
| **By a non-letter** | **14** | *9* | **12** | *12* |
| **ADDITION** | **7** | *4* | **2** | *2* |
| **TRANSPOSITION** | **4** | *3* | – | – |
| Exchange | **4** | *3* | | |
| Shift | – | – | – | – |
| **DELETION** | **9** | *6* | **7** | *7* |
| **MULTIPLE-LETTER ERROR** | **6** | **4** | **10** | **10** |
| **OTHERS** | **14** | **9** | **8** | **8** |
| ORTHOGRAPHIC | 6 | 4 | 1 | 1 |
| RESTART | 7 | 4 | 4 | 4 |
| AMBIGUOUS | 1 | 1 | 3 | 3 |
| **TOTAL ERRORS** | **157** | **100** | **103** | **100** |

and oral spelling. In both tasks, AD's error distribution differed strikingly from that of the control subjects (Fig. 4). In particular, in comparison with the control subjects, AD produced a higher proportion of letter substitution errors in written spelling (AD: 71% vs. controls: 22%) and a higher proportion of letter deletion (AD: 36% vs. controls: 7%) and multiple-letter errors (AD: 21% vs. controls: 11%) in oral spelling; in both tasks, AD produced instead a smaller proportion of orthographic errors than the control subjects (AD: 8% and 9% vs. controls: 63% and 34%, in written and oral spelling, respectively). This finding of qualitative differences between the errors produced by AD and the controls undermines an account of the mild impairment observed in AD's spelling performance merely in terms of a non-specific effect of brain damage and strengthens the view that, in spite of relatively good spelling accuracy, AD did have damage to spelling processing component(s).

*3.1.4.2. Error rates as a function of letter position*

Only single-letter errors were included in this analysis. We used the procedure proposed by Wing and Baddeley [52] to normalize the distribution of errors across the various stimulus length. According to this procedure, each stimulus is divided into five positions: A, B, C, D, E. Each position contains one letter or more, depending on the number of letters that exceeds five or multiples of five in the stimulus. The letters in excess are distributed across the five positions so as to maintain a symmetrical structure in this reconstructed stimulus.[2] Then, each position was assigned one point or half a point per error, following the procedure described by Caramazza and Miceli [8]: (i) the position involved in a letter substitution or deletion error was assigned one point; (ii) in the case of a letter addition, half a point was assigned to each position adjacent to the added letter; (iii) in a letter exchange error, both letter positions involved were assigned one point; (iv) for letter shifts, the position which the letter was moved from was assigned one point and the position to which the letter moved to was scored like a letter addition error.

Figure 5 displays the combined serial position curve for written spelling, oral spelling, and typing. A contrasting distribution of error positions appears across the three tasks ($\chi^2_{(8)} = 26.14$; $p < 0.001$), with the distribution noted in written spelling being significantly different from both the distribution noted in oral spelling ($\chi^2_{(4)} = 22.33$; $p < 0.0001$) and typing ($\chi^2_{(4)} = 13.81$; $p < 0.01$), whereas the distribution noted in oral spelling and typing did not significantly differ one from another ($\chi^2_{(4)} < 1$). In oral spelling, the error rate was higher in the middle positions B and C than in the positions A, D, E (for all comparisons between B and C and the other positions: $\chi^2_{(1)} > 15$; $p < 0.0001$; for all comparisons between A, D, and E: $\chi^2_{(1)} < 1$). The same pattern was found in typing, with more errors in the middle positions B and C than in positions A, D, E (for all comparisons between B and C and the other positions: $5 < \chi^2_{(1)} < 13.5$; $0.01 < p < 0.001$; for all comparisons between A, D, and E: $0.2 < \chi^2_{(1)} < 1.3$; $0.27 < p < 0.65$). Thus, both in oral spelling and typing, letters located at the middle of the stimuli were subjected to more errors than letters at the initial or final positions – a pattern that strongly suggests the involvement of a memory component at the processing level affected by the deficit. This pattern was not found in written spelling, where the error rates were not significantly different across stimulus positions ($0.5 < \chi^2 < 2.9$ and $0.24 < p < 0.95$), with the exception of the error rate in the final position, which was smaller than the error rate in the middle position $C(\chi^2_{(1)} = 4.61$; $p < 0.05$).

In sum, the results show that AD's errors in oral spelling and typing exhibit the characteristic features of errors resulting from damage to the graphemic buffer: errors tended to increase with the stimulus length and mostly affected the letters at the middle positions within the stimuli. Also, AD produced a high rate of letter deletion and multiple-letter errors in oral spelling, and previous studies have shown that those particular kinds of letter errors were strongly correlated with the severity of damage to the graphemic buffer [8]. None of these features was found in the errors AD produced in written spelling and, in addition, one particular type of letter error – noncontextual letter substitution errors – was frequently noted in written spelling and virtually absent in oral spelling (and typing).

*3.1.4.3. Features of noncontextual letter substitution errors*

Since noncontextual letter substitution errors were found in AD's written spelling and not in oral spelling (and typing), one can put forward the hypothesis that they were caused by damage to a mechanism specifically dedicated to written spelling. If this hypothesis is correct, these errors should not show the same characteristics as the letter errors found in oral spelling. In particular, assuming that noncontextual letter substitution errors in written spelling reflects damage to letter form processing, and given that there is no theoretical reason to assume that the mechanisms at that level have a memory component, these errors are not expected to show the typical distribution of error position found in oral spelling. Figure 6 shows the serial position curves for the noncontextual letter substitutions (N = 45) produced in written spelling and the letter errors (single- and multiple-letter errors) produced in oral spelling (N = 131). Both distributions were significantly different ($\chi^2_{(4)} = 23.65$; $p < 0.0001$) and this difference

[2] Stimuli of three and four letters were also included in this analysis (161 in written spelling, 159 in oral spelling, and 136 in typing). The letters of the three-letter stimuli were assigned the positions A, C, E and the letters of the four-letter stimuli, the positions A, B, D, E.

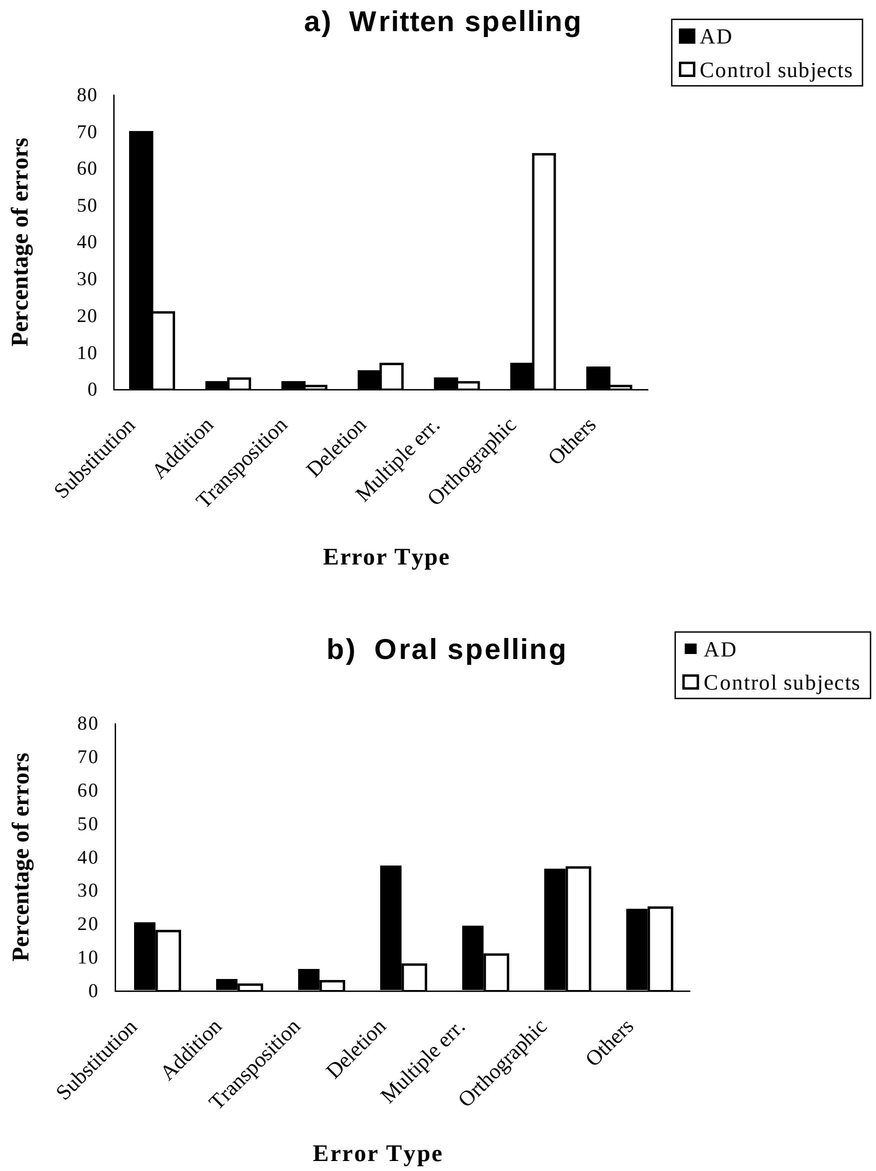

Fig. 4. Distribution of the various error types made by AD and the control subjects in written spelling (a) and in oral spelling (b). (Note: Here, the error type "Others" refers to restart and ambiguous errors).

was due to the noncontextual letter substitution errors occurring significantly more often at the first stimulus position ($\chi^2_{(1)} = 14.69$; $p < 0.0001$) and less often at the last position ($\chi^2_{(1)} = 3.87$; $p < 0.05$) than the letter errors found in oral spelling (the occurrence of both kinds of errors at the other stimulus positions was not significantly different).

Moreover, it has been found that patients with damage at a post-graphemic level of the writing processes tend to substitute target letters with more frequent letters [4]. We looked at the frequency[3] of the target and erroneous letter in the noncontextual letter substitution errors produced in written spelling and the letter errors produced in oral spelling. We found that the erroneous letter was of higher frequency than the target letter in 75% of the noncontextual letter substitution errors produced in written spelling and in only 53% of the letter

[3] In this analysis, we considered the frequency of occurrence of each letter in the 31 million word corpus of Frantext (http://www.lexique.org).

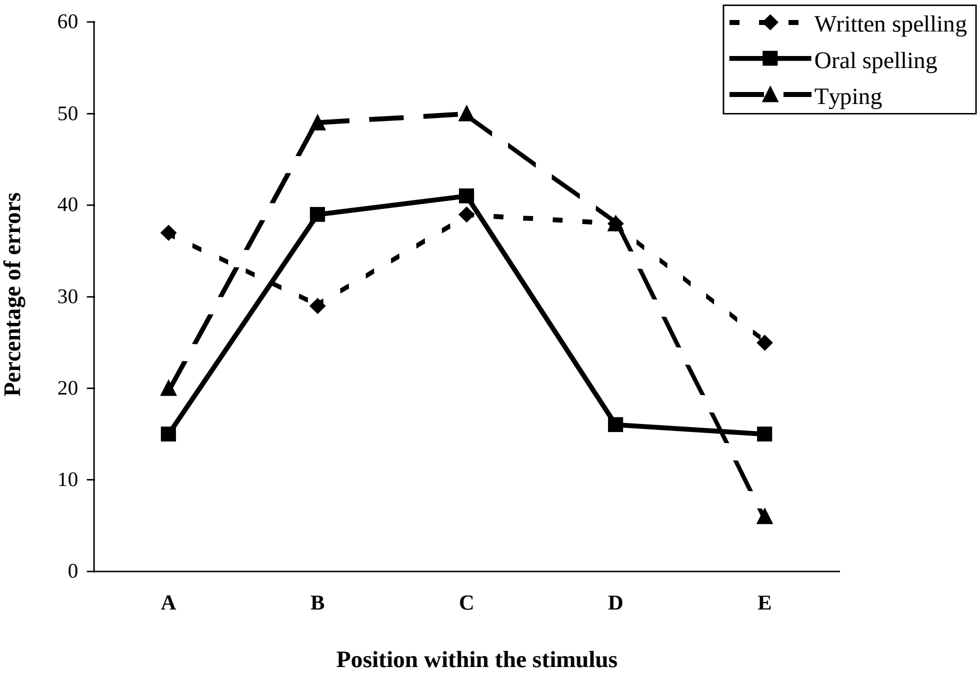

Fig. 5. Distribution of AD's single-letter errors across five stimulus positions, in written spelling, oral spelling, and typing.

errors produced in oral spelling, a significant difference ($\chi^2_{(1)} = 4.12$; $p < 0.05$).

### 3.2. Interim summary and discussion

The analysis of AD's spelling performance indicated that he was impaired in the three modalities of spelling output, that is, in written spelling, oral spelling, and typing and that his error rate was also similar in the three tasks. Moreover, in the three tasks, AD's level of performance was unaffected by the stimulus being a familiar word or a nonword, nor by lexical variables like word frequency, imageability, or grammatical class. These aspects of AD's pattern of performance point to a deficit located at a post-lexical level of processing within the functional architecture of the spelling system. Given that most of AD's errors in the three tasks were letter-level errors, the most straightforward hypothesis regarding the damaged processing level responsible for the impaired performance in the three tasks is the graphemic buffer.

However, aspects of AD's pattern of performance are not consistent with this hypothesis. First, compared to the controls, AD's performance in written spelling was more impaired than his performance in oral spelling. Second, the two hallmarks of damage to the graphemic buffer – i.e., the increase of letter error rates with stimulus length and the relative increase of error rates at the middle positions of the stimulus – were noted in oral spelling and typing only, not in written spelling. Third, the distribution of the letter error types differed between written and oral spelling and typing. In particular, one error type, the noncontextual letter substitution errors, was frequent in written spelling and virtually absent in oral spelling and typing. Fourth, some instances of distorted letters were observed in written spelling, which suggests at least mild damage to writing-specific processes.

From this contrasting pattern of performance and error distribution in written and oral spelling and typing, we propose that AD's impaired performance in these tasks resulted from a combination of distinct deficits. We mentioned earlier the two most plausible combinations of deficits that could be entertained. Now we must reject the first one, namely, the hypothesis that AD's impaired performance in three spelling tasks resulted from damage to each of the modality-specific mechanisms involved in written spelling, oral spelling and typing. We indeed found converging pieces of evidence that AD's impaired performance in oral spelling and typing resulted from damage to a post-lexical component of the spelling system that is shared by both modalities of output. AD's performance was similarly impaired in both tasks in comparison with the con-

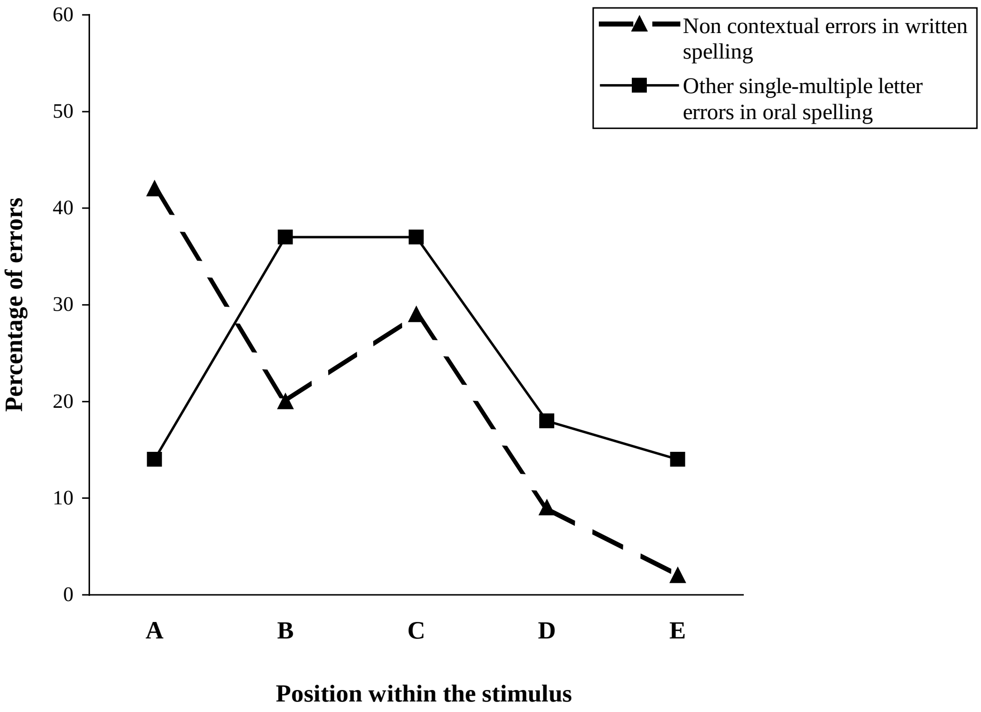

Fig. 6. Distribution of AD's noncontextual letter substitution errors produced in written spelling and letter-level errors produced in oral spelling across five stimulus positions.

trols' performance. In both tasks, the patient produced mostly letter-level errors which exhibited the characteristic features of errors resulting from damage to the graphemic buffer: errors tended to increase with stimulus length and mostly affected middle positions within the stimuli. Thus, results indicate that AD had damage at the level of the graphemic buffer, which impaired his performance in oral spelling and typing, and thus also, according to our model, in written spelling. In order to account for contrasting patterns of AD's performance in oral spelling and typing on the one hand, and written spelling on the other, additional damage to a writing-specific component has to be assumed, which further impaired performance in written spelling only. Given the type of errors produced in written spelling, this damaged component is most probably a component dedicated to letter form processing.

Furthermore, we found evidence that the noncontextual letter substitution errors produced by AD in written spelling did not show the features expected from errors resulting from damage to the graphemic buffer. We propose that the noncontextual letter substitution errors produced by AD in written spelling originate from damaged mechanisms distinct from those causing other letter errors in oral spelling and typing. These other errors (i.e., contextual letter substitutions, letter additions, deletions, and transpositions and multiple-letter errors) would mainly reflect a deficit in the mechanisms assigning position to the graphemes held in the graphemic buffer. Because these mechanisms are common to all modalities of spelling output, these letter errors were found both in AD's written and oral spelling and typing. As for noncontextual letter substitution errors, they would reflect a deficit in the mechanisms that assign form to graphemes during written spelling. Admittedly, noncontextual errors might *theoretically* originate from damage to the graphemic buffer, since that level is supposed to hold information about grapheme identity, not only position within a word. Thus, a noncontextual letter substitution could arise from information about the identity of a given grapheme being lost and replaced by an incorrect one. However, such an account could not explain why this kind of error was virtually absent from AD' errors in oral spelling (and typing). Finally, we suggest that the few distorted letters produced by AD in written spelling could be ascribed to the same impaired mechanisms as those causing noncontextual letter substitution errors. Whereas a noncontextual letter substitution would result from a failure in selecting the appropriate letter form among similar letter forms, a distorted letter would reflect incomplete retrieval of the form of a given grapheme or blending of two similar letter forms.

A possible alternative account for the noncontextual letter substitution errors produced by AD needs to be addressed. In fact, these errors might originate from perseverations of letters occurring in the preceding trials of the stimulus list. Therefore, we examined the 45 noncontextual letter substitutions produced in written spelling in relation to letters included within the three immediately preceding trials (trials N-1, N-2 and N-3) and the sixth preceding trial (trial N-6) [50]. According to the perseveration account, the number of erroneous letters identical to letters included in preceding trials should be the highest when the immediately preceding trials (trial N-1) are considered and it should progressively decrease when the other previous trials are examined. This pattern was not found: 38%, 40%, 38% and 42% of the erroneous letters in noncontextual letter substitution errors were present in trials N-1, N-2, N-3 and N-6, respectively. Thus, it is unlikely that noncontextual letter substitution errors produced by AD in written spelling were perseverations of letters he wrote in previous items of the stimulus list.

Having reached a motivated hypothesis about the processing levels of the spelling system that are damaged in AD, the next step is to address the issue of the kinds of letter representation that are processed at these levels. In this perspective, we examined the kind of relationship between target and error in AD's letter substitution errors.

### 3.3. *Analyses of the target-error relationship in AD's letter substitution errors*

According to our hypothesis, the noncontextual letter substitution errors produced by the patient in written spelling originated from damage to the component assigning form to graphemes. On the other hand, the other letter errors, noted both in written and oral spelling, result from damage to the graphemic buffer. As we mentioned in the Introduction, distinct kinds of letter representation are assumed at these levels: amodal, symbolic letter representations at the level of the graphemic buffer, and modality-specific representations of letter form at the subsequent level of the writing processes. On the basis of these assumptions, one can predict that if two letter representations were confused during processing at the level of the (damaged) graphemic buffer, then these two letters should bear no physical similarity, since letter representation, at this level, is assumed to specify grapheme identities, without reference to their name, shape, or font (symbolic representation of letter identity). On the other hand, if two letter representations were confused during processing at the level of a (damaged) component assigning form to graphemes, these two letters should be similar in form, that is, "physically" similar. Therefore, if the assumption of multiple and distinct representations of letter information is correct, we should observe distinct patterns of similarity within the letter substitution errors produced by AD: only in the noncontextual letter substitution errors produced in written spelling should the target and erroneous letter be physically similar. Then, the question raised is whether "physical" similarity would be in terms of visuospatial or stroke feature similarity. Exploring this question should reveal whether the letter form information represented at the writing-specific processing level that is damaged in AD provides a description of the shapes of letters in terms of their visual features or of their form in terms of the characteristics of the graphic strokes required to produce the specific form.

We addressed these issues by examining the nature of the similarity between the target and erroneous letters in the letter substitution errors produced by the patient in written and oral spelling (typing errors were not analysed). For the purpose of this analysis, we developed both a visuospatial and a stroke feature similarity metrics on the basis of the patient's own handwriting.

#### 3.3.1. *Method*

##### 3.3.1.1. *The visuospatial similarity metric*

Instead of using a standard visual confusion matrix [21], we developed a visual similarity metric based on the patient's own handwriting. Thus, he was asked to write successively and in lowercase script the 26 letters of the alphabet on a sheet of paper. These 26 written letters were then arranged by pairs (i.e., 325 letter pairs) and subdivided into two sublists. The letter pairs were then presented horizontally on a cardboard, one pair at a time, to two groups of 30 subjects (one group per sublist), who were asked to rate each letter pair on overall visual similarity, with "1" representing low visual similarity and "5" high visual similarity. On the basis of these ratings, a visual similarity matrix was prepared, which provides the mean rated value for each of the 325 letter pairs. We set the cut-off for visual similarity at a mean rated value $\geqslant 2.8$; this criterion was reached for 33 letter pairs (10%).

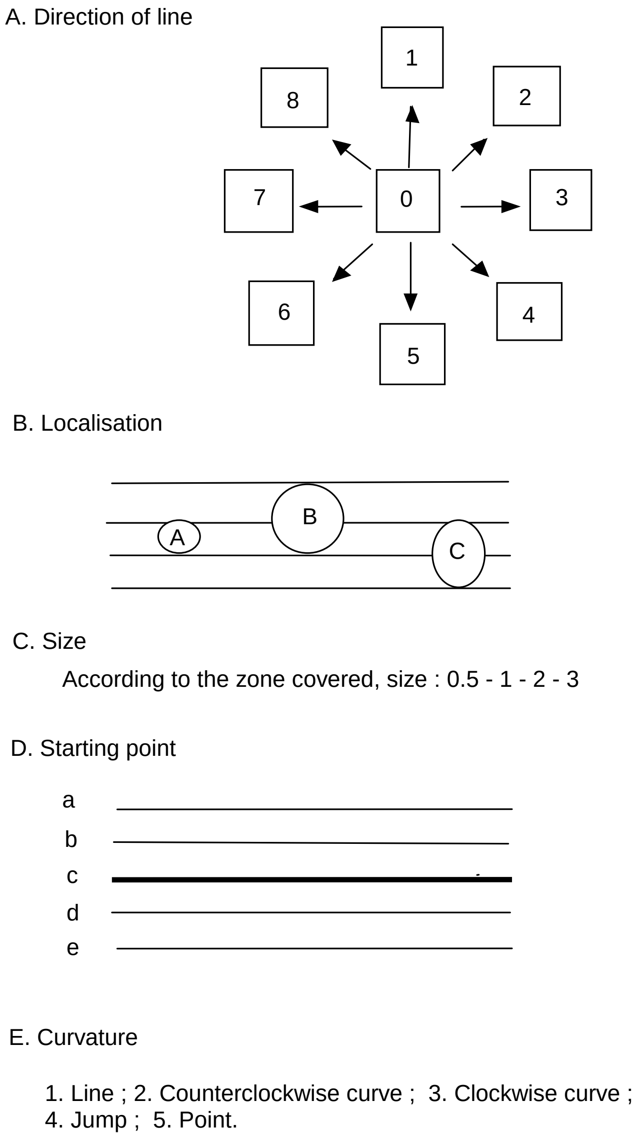

Fig. 7. The five features used to describe each component stroke of each letter, with their various possible values.

*3.3.1.2. The stroke feature similarity metric*

The patient was asked to write in lowercase script, successively and in isolation, the 26 letters of the alphabet on a digitising tablet. Each written letter was then decomposed into its component strokes, according to Meulenbroeck and Van Galen's criteria [33]. Thus, a stroke was defined as a written segment between two direction changes. "Invisible" strokes, that is, movements in the air that are part of the sequence of gestures required to produce the letter were also considered. This procedure resulted in letters being decomposed into 3, 4, 5, or 6 strokes. Letter *l*, for instance, was decomposed into three strokes (see Table 7).

In the next step, each component stroke of each letter was described according to five characteristics (see Fig. 7): direction of line, localization, size, starting point, and curvature (adapted from Rapp and Caramazza [40]). In this way, 43 distinct strokes were identified and then assigned a given label (using uppercase letters). For example, the first stroke of *l* was described as follows: direction of lines = 2, localization = AB, size = 2, starting point = c, and curvature = counterclockwise curve, and labelled "ZK" (the labels assigned to the component strokes of each of the 26 letters of the alphabet are given in Appendix A).

Table 7
The three strokes identified in the letter $l$ produced by AD

| Stroke 1 | Stroke 2 | Stroke 3 |
|---|---|---|

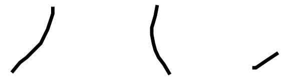

Finally, in order to draw a stroke feature similarity matrix for all possible letter pairs (N = 325), the sequence of component strokes of each letter was compared with that of every other letter in the alphabet and the number of strokes (from 0 to 4) shared by each letter pair, irrespective of whether they were in the same position, was counted. For example, letter $l$ has the following sequence of strokes: "ZK-A-L" and letter $b$ the following: "ZK-A-E-P". They thus shared two strokes. Letter pairs were deemed to be similar in terms of stroke features if they shared 2, 3, or 4 strokes. This was the case for 32 letter pairs (10%): 27 pairs (8%) shared 2 strokes, 2 pairs shared 3 strokes, and 3 pairs, 4 strokes.

*3.3.1.3. Scoring similarity of target-error letter pairs*

Following Rapp and Caramazza [40], we considered four categories of similarity between letter pairs: a) Visuospatially similar only: when the target and error had a value of visuospatial similarity $\geqslant$ 2.8 and no stroke in common; b) Similar in stroke features only: when target and error shared at least two strokes and had no visuospatial similarity ($\leqslant$ 1.5); c) Ambiguous: when target and error both had a value of visuospatial similarity $\geqslant$ 2.8 and shared at least two strokes; d) Other pairs: this category includes unrelated pairs (the target and error had a value of visuospatial similarity $\leqslant$ 1.5 and no stroke in common) and unclassifiable pairs (the target and error had a value of visuospatial similarity included in 1.6 to 2.7 and 0 or 1 stroke in common). These criteria resulted in all possible letter pairs characterized as displayed in Table 8. On this basis, we then classified each target-error pair noted in the letter substitution errors produced by AD in written and oral spelling in one of the four categories of similarity.

*3.3.2. Results*

The list of all target-error pairs noted in AD's letter substitution errors, classified into the various categories of similarity, are displayed in Appendix B. The results (summarized in Table 9) revealed that in written and oral spelling the majority of target-error pairs in AD's letter substitution errors were "other pairs" (i.e., unrelated or unclassifiable). Importantly, no target-error pair was observed that was "visuospatially similar only". Moreover, a first data analysis revealed that the distribution of target-error pairs across the four categories of similarity significantly differed between the noncontextual letter substitutions and the contextual letter substitutions produced in written ($\chi^2_{(3)} = 14.21$; $p < 0.0001$) or oral spelling ($\chi^2_{(3)} = 5.81$; $p = 0.05$), whereas the distribution of target-error pairs did not significantly differ between the contextual letter substitutions produced in written and oral spelling ($\chi^2_{(3)} = 1.41$; $p = 0.49$). Decomposing the significant effect indicated that (i) the rate of "other pairs" was significantly lower in the noncontextual errors than in the contextual errors produced in written ($\chi^2_{(1)} = 11.87$; $p < 0.001$) or oral spelling ($\chi^2_{(1)} = 4.71$; $p < 0.05$); (ii) there were significantly more "ambiguous" target-error pairs in the noncontextual errors than in the contextual errors produced in written ($\chi^2_{(1)} = 5.55$; $p < 0.02$) or oral spelling ($\chi^2_{(1)} = 3.74$; $p = 0.05$); (iii) there were also more target-error pairs "similar in stroke features only" in the noncontextual errors than in the contextual errors produced in written spelling ($\chi^2_{(1)} = 3.71$; $p = 0.05$) but not in oral spelling ($\chi^2_{(1)} < 1$); (iv) when the categories "similar in stroke features only" and "ambiguous" were merged, a reliable difference was observed between the noncontextual errors and the contextual errors produced in written spelling ($\chi^2_{(1)} = 11.87$; $p < 0.0001$) or oral spelling ($\chi^2_{(1)} = 4.71$; $p < 0.05$), although no significant difference emerged between the contextual errors produced in written and oral spelling ($\chi^2_{(1)} = 1.41$; $p = 0.49$).

Furthermore, we evaluated the possibility that the observed rates of target-error pairs in each category of similarity could have resulted from a mere random association of target and erroneous letters (see [40]). For this purpose, each target and error letter was randomly re-paired 1,000 times and the resulting target-error pairs then classified in one of the four categories of similarity. The results (see Fig. 8) indicated that for the contextual errors produced both in written and oral spelling, the observed rates of target-error pairs that were similar in "stroke features only" lie within the chance region. On the other hand, for the noncontextual errors, the observed rates of target-error pairs that were similar in "stroke features only" were never generated in 1,000 random re-pairings of the target and error letters. Moreover, for the noncontextual errors, the observed rates of pairs classified as ambiguous were far outside the chance region, whereas for the noncontextual errors, they only just exceeded the chance level rates.

Table 8
Distribution of all possible letter pairs across the four categories of similarity

| Category of similarity | Letter pairs (N = 325) |
|---|---|
| Visuospatially similar only | 13 letter pairs (4%)  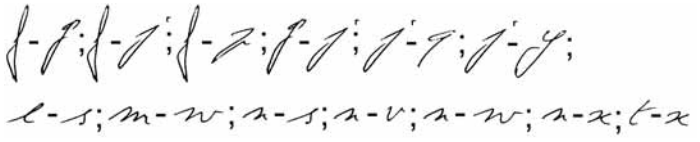 |
| Similar in stroke features only | 11 letter pairs (3%)  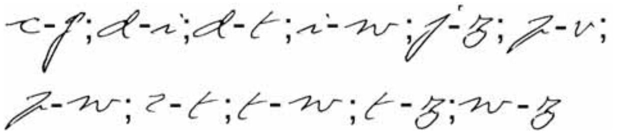 |
| Ambiguous | 9 letter pairs (3%)  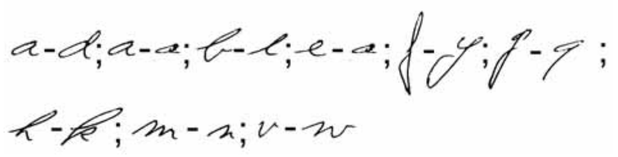 |
| Other pairs | 292 letter pairs (90%) |

Table 9
Distribution of the letter substitution errors produced by AD in written and oral spelling, across the four categories of target-error pair similarity

| TYPE OF SUBSTITUTION ERROR | Visuospatially similar only | Similar in stroke features only | Ambiguous | Others |
|---|---|---|---|---|
| **Written spelling** | | | | |
| Noncontextual substitution errors ($N = 45$) | 0% | 13% | 36% | 51% |
| Contextual substitution errors ($N = 58$) | 0% | 2% | 14% | 84% |
| **Oral spelling** | | | | |
| Contextual substitution errors ($N = 32$) | 0% | 6% | 16% | 78% |

### 3.3.3. *Discussion*

In sum, in spite of the relatively small corpus of errors included in both the data analyses above, the results converge in showing distinct patterns of similarity between target and error within the letter substitution errors produced by AD. In particular, the noncontextual letter substitution errors bore a physical similarity to the intended target significantly more often than the contextual letter substitutions produced in both written and oral spelling. That physical similarity was based, at above chance levels, on features of the graphic strokes composing the letters rather than visuospatial features. Moreover, we found no target-error pair similar in terms of visuospatial features only, whether in the noncontextual or in the contextual errors. On this basis, we suggest that the above chance level proportion of "ambiguous" target-error pairs found in the noncontextual errors were likely genuine stroke-feature similar pairs which happen, by chance, also to be visuospatially similar. As for the contextual errors produced by AD, the results indicated that they present a similar pattern whether they were produced in written or oral spelling and, in particular, that the erroneous letters bore no physical similarity to their targets. Given the hypothesis we put forward about the distinct origin of the noncontextual and the contextual errors, these results support the distinction between symbolic letter representation at the level of the graphemic buffer and letter representation in terms of the component letter strokes at the level of letter form processing.

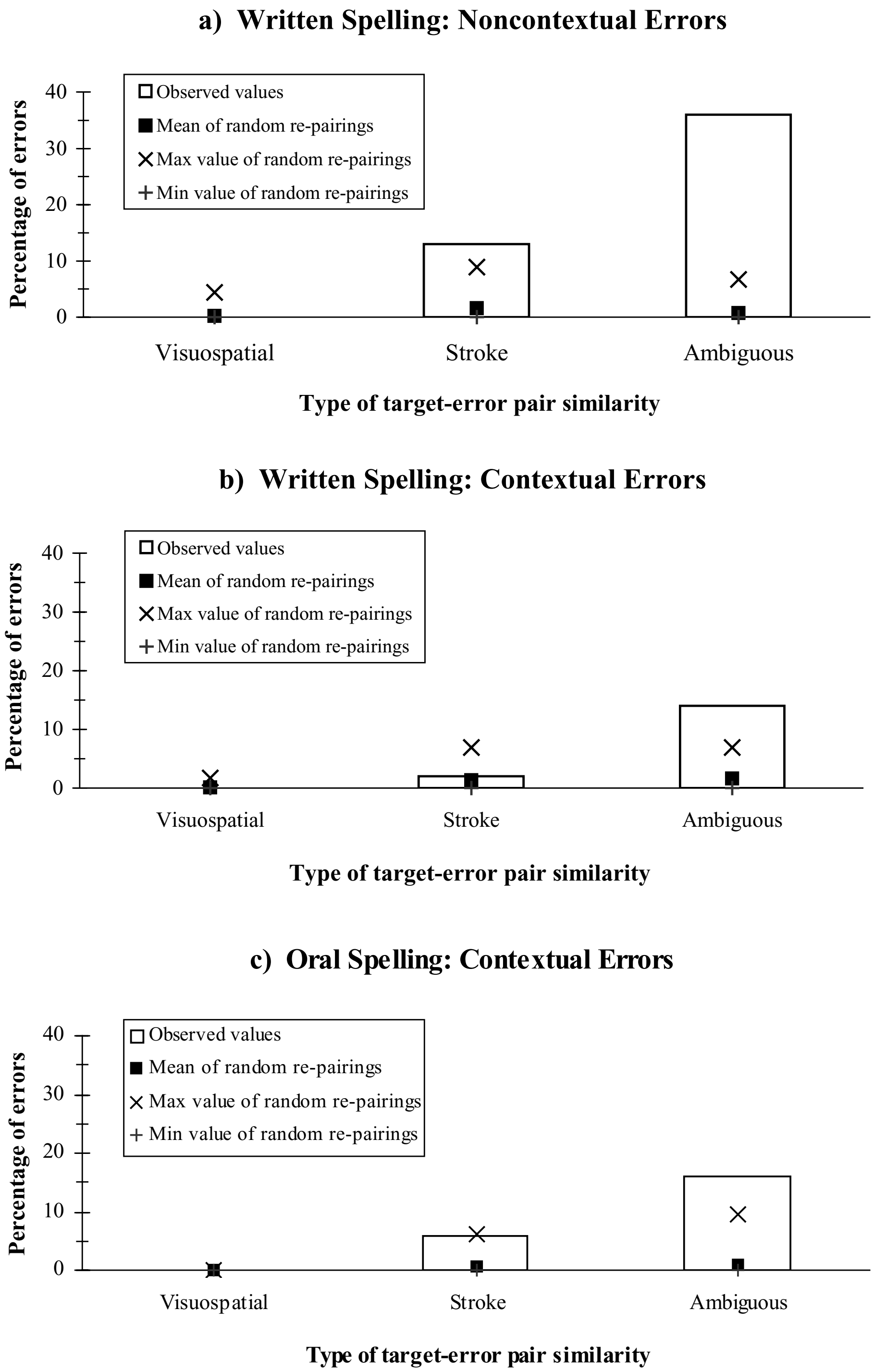

Fig. 8. Observed and randomly generated values for the various categories of similarity between target and error letter, for the noncontextual letter substitution errors (a) and the contextual letter substitution errors produced in written spelling (b) and the contextual letter substitution errors produced in oral spelling (c).

### *3.4. Additional analyses of AD's written spelling errors*

In this section, we provide additional details about the pattern of letter substitution errors produced by AD in written spelling. One striking feature is that some letters are particularly vulnerable in the noncontextual letter substitution errors, namely, the vowel $o$ substituted by $a$ and the consonants $t$ and $d$ substituted one by the other (see Appendix B). In the "$o - a$" target-error pair, AD tended to produce the stroke "ZG" before the stroke "Y", shared by both letters. When he had to produce $d$ (substituted by $t$), he did not produce the first stroke "Y". It is worth noting that these target-error pairs were never observed when AD wrote in uppercase script. In that case, the more frequently involved letters were the consonants $N$ and $L$, which were substituted one by the other, and $T$ substituted by $L$. Thus, AD encountered particular difficulties with some letters, and not the same letters when writing in lowercase and uppercase, which suggests that his writing difficulties were genuinely related to the specific sequence of the strokes composing the letter form to be produced.

Second, it appeared that the nine instances of contextual errors being similar in terms of strokes to the intended letters ("stroke-feature similarity only" and "ambiguous") all concerned the letters that were the most often confused in the noncontextual errors (that is, $o$ and $d$). This might reflect the inherently ambiguous nature of the errors scored as "contextual". Thus, a number of them could have resulted in fact from the same error mechanism than the one giving rise to the noncontextual errors. In an unknown number of cases, it might happen, just by chance, that a letter substituted by that mechanism occurred elsewhere in the word. In other words, one cannot rule out that a number of errors scored as "contextual letter substitutions" in written spelling were in fact caused by damage to the letter form processing level, like the errors scored as "noncontextual letter substitutions", while others reflected damage to the graphemic buffer.

That some letters appeared particularly vulnerable in written spelling might (at least partly) account for the relatively higher rate of letter substitution errors noted at the beginning of the words in written spelling (in comparison with the rate of letter substitution errors in that position in oral spelling and typing). Indeed, we found that the letters $o, d, t, j$ and $v$, which were involved in 49% of the noncontextual letter substitution errors, were not evenly represented across all stimulus positions within the word lists presented to AD in written spelling (total words = 1,159). As shown in Fig. 9, vulnerable letters appeared more often in position A and C than in the positions B, D and E (A-B: $\chi^2_{(1)} =$ 13.93; $p <$ 0.0002; A-D: $\chi^2_{(1)} =$ 67.13; $p <$ 0.0001; A-E: $\chi^2_{(1)} =$ 35.06; $p <$ 0.0001; but A-C: $\chi^2_{(1)} <$ 1).

## 4. General discussion

In this study, we examined the case of a patient whose performance in written spelling, oral spelling, and typing was mildly impaired. We showed that his pattern of errors in oral spelling and typing was consistent with the hypothesis that errors were caused by damage to the graphemic buffer, i.e., a working memory component that holds information about grapheme identity and position during output processing. Furthermore, we found evidence that impaired performance in written spelling reflected additional damage to a writing-specific component, i.e., a component assigning form to graphemes.

Evidence of additional damage to a writing-specific component mainly stemmed from the distinction we drew between contextual and non contextual letter substitutions in the classification of the patient's errors. To the best of our knowledge, this distinction has not been considered in previous studies of dysgraphic patients.[4] Thus, one question is to what extent neglect of this distinction may have been responsible for the contradictory patterns in the performance of patients that were all supposed to have damage to the graphemic buffer [11]. For example, when it turned out that the patient's performance in written spelling did not conform to the pattern expected in case of damage to the graphemic buffer (e.g., as regards the effect of stimulus length or the distribution of the errors across stimulus positions), it is an open question whether drawing a distinction between contextual and noncontextual letter substitution errors would help to resolve the inconsistencies. In the present case study, at least, this distinction did help understand the origin of apparent inconsistencies and, eventually, of the patient's errors in written spelling.

[4]The sole exception we noted is in the paper of Rapp and Caramazza [40]. In a footnote (p. 1137), these authors mentioned that, for the analysis of the error types produced by a patient with an impairment specific to writing, they put together both kinds of errors into a single category of "substitution", because "separate analyses on the different types revealed no difference". However, they did not report the relative proportion of each error type.

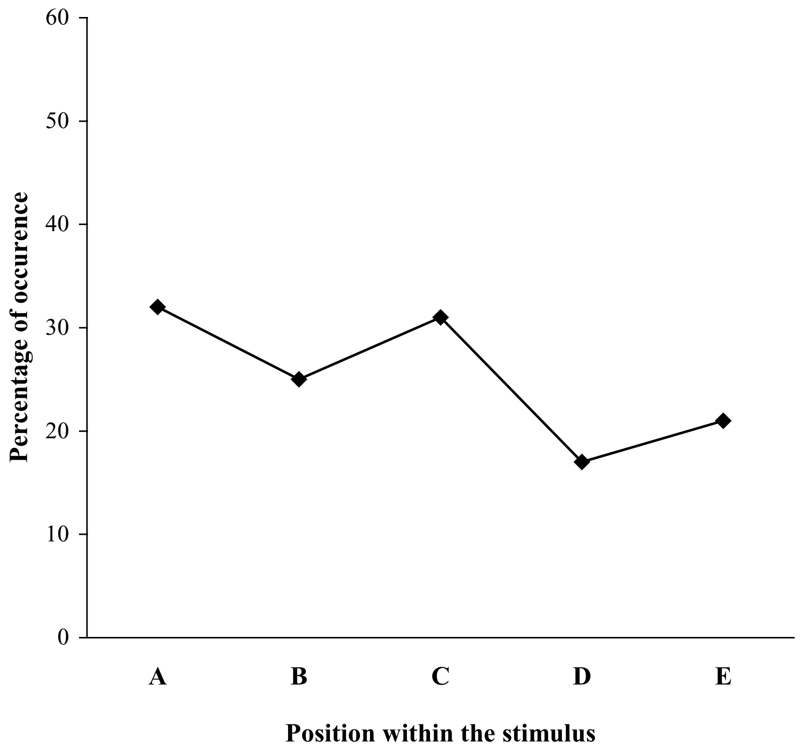

Fig. 9. Combined distribution of letters $o, d, t, j$, and $v$ across various stimulus positions in the 1159 word stimuli presented to AD in written spelling.

Once evidence was found of damage to two distinct levels of letter processing within the spelling system, we sought evidence for the hypothesis that distinct types of letter representation are processed at each of these damaged levels – symbolic letter representations at the graphemic level and representations of letter strokes at the letter-form level. On the basis of a visuospatial and a stroke-feature similarity metrics developed from patient's own handwriting, we found that the noncontextual letter substitution errors produced in written spelling were similar to the target letters in terms of component strokes. This is consistent with the pattern of similarity found between target and error in two cases described by Rapp and Caramazza [40], JGE and HL, who had impairment specific to written spelling. Their patients showed letter substitution errors in writing uppercase letters that were similar in component strokes to the targets. On the other hand, for the contextual letter substitution errors produced by our patient in written and oral spelling, we found no physical similarity between target and error. This result is consistent with the analysis of letter substitution errors produced in written spelling by two other patients described by Rapp and Caramazza [40], LB and HE, whose pattern of impaired performance in written and oral spelling pointed to damage to the graphemic buffer: in these cases, no physical similarity was found between target and error neither. Thus, in the context of a single case study, we found evidence that converges with evidence from patients with distinct deficits, supporting the assumption that letter information processed at the graphemic level and at the letter-form processing level are of distinct types.

Let us make clear, however, that we are not arguing that contextual and noncontextual letter substitution errors could be taken as reliable and direct reflections of damage to the graphemic buffer or to a writing-specific component, respectively. As mentioned earlier, noncontextual errors could also be expected from damage to the graphemic buffer, when information about grapheme identity has been lost or confused and, on the other hand, errors scored as contextual might well be instances of errors originating from mis-selection of the appropriate stroke sequence for a target letter. In our view, both kinds of error can be given a reliable interpretation only by reference to the patient's performance and error distribution across the different modalities of spelling output.

## Acknowledgements

This paper is dedicated to AD, who died in April 2005. We are grateful to Brenda Rapp for her insightful comments and recommendations on this case study. We also owe thanks to Adam Buchwald for having very kindly provided us with the program for randomly pairing letters. Thanks to Marie Van Reybroeck and Delphine Guitton for their assistance in testing the control subjects, Eva Turconi for her help in statistical analyses, and Doctor Adrian Ivanoiu for his advice on interpreting the medical records of the patient. We are also grateful to Brendan Weekes and two anonymous reviewers for their useful suggestions on an earlier version of this paper.

**Appendix A**
Sequence of the component strokes for the 26 letters produced by AD in lowercase script. The capital letter(s) is (are) a label assigned to a given stroke after having been described according to five features (direction, localization, size, starting point, and curvature of the stroke; see text)

| Letter | Stroke 1 | Stroke 2 | Stroke 3 | Stroke 4 | Stroke 5 | Stroke 6 |
|---|---|---|---|---|---|---|
|  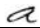 | ZG | Y | E | Q | L | |
|  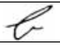 | ZK | A | E | P | | |
|  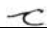 | G | Y | J | ZE | L | |
|  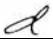 | Y | ZJ | B | L | | |
|  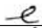 | M | ZF | U | L | | |
|  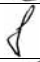 | ZK | C | ZN | ZP | | |
|  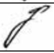 | G | Y | F | ZL | ZR | |
|  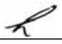 | ZK | B | K | Q | | |
|  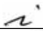 | O | Y | L | ZH | D | |
|  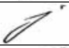 | O | ZM | ZO | ZH | D | |
|  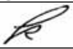 | ZK | B | J | ZB | T | |
|  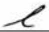 | ZK | A | L | | | |
|  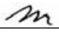 | N | Z | N | Z | N | Z |
|  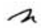 | N | Z | N | Z | | |
|  | Y | M | ZF | Q | | |
|  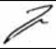 | O | ZL | ZQ | P | | |
|  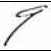 | Y | F | ZL | | | |
|  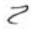 | I | P | ZA | L | | |
|  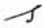 | O | W | X | ZC | ZF | |
|  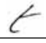 | B | L | ZI | P | | |
|  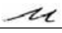 | O | U | M | Y | | |
|  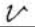 | H | V | O | P | | |
|  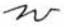 | H | V | L | R | O | P |
|  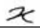 | H | ZD | O | W | S | |
|  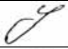 | U | M | ZL | ZN | ZP | |
|  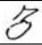 | I | P | ZA | L | ZM | ZO |

**Appendix B**

List of all target-error letter pairs noted in AD's letter substitution errors according to the category of similarity between letter pairs (the number of occurrences of each target-error letter pair is given in parentheses)

| | CATEGORY OF SIMILARITY BETWEEN LETTER PAIRS | | | | |
|---|---|---|---|---|---|
| TYPE OF SUBSTITUTION ERROR | Visuospatially similar only | Similar in stroke features only | Ambiguous | Unrelated | Unclassifiable |
| Noncontextual substitution errors | – | d-t (2) | o-a (15) | b-s (1) | a-u (1) |
| produced in written spelling | | j-z (1) | v-w (1) | o-r (1) | b-f (2) |
| ($N = 45$) | | t-d (3) | | p-c (1) | b-t (1) |
| | | | | p-l (1) | c-o (1) |
| | | | | y-i (2) | d-l (1) |
| | | | | | g-n (2) |
| | | | | | i-u (3) |
| | | | | | l-t (1) |
| | | | | | s-j (1) |
| | | | | | s-r (2) |
| | | | | | u-i (1) |
| | | | | | y-u (1) |
| Contextual substitution errors | – | d-t (1) | o-a (5) | a-p (1) | a-b (1) |
| produced in written spelling | | | o-e (3) | b-e (1) | a-e (3) |
| ($N = 58$) | | | | b-s (2) | a-h (1) |
| | | | | e-n (1) | a-t (1) |
| | | | | g-a (3) | b-f (1) |
| | | | | h-r (2) | b-p (2) |
| | | | | m-g (1) | b-r (1) |
| | | | | o-t (1) | c-t (3) |
| | | | | t-g (1) | d-g (1) |
| | | | | u-l (1) | e-l (2) |
| | | | | y-i (1) | e-r (1) |
| | | | | | h-l (2) |
| | | | | | i-e (1) |
| | | | | | i-r (1) |
| | | | | | i-f (1) |
| | | | | | l-o (1) |
| | | | | | n-i (2) |
| | | | | | p-t (1) |
| | | | | | r-n (1) |
| | | | | | r-v (1) |
| | | | | | t-a (1) |
| | | | | | t-b (1) |
| | | | | | t-l (1) |
| | | | | | t-p (1) |
| | | | | | u-i (1) |
| | | | | | v-s (1) |
| Contextual substitution errors | – | c-g (1) | e-o (1) | e-n (1) | b-p (1) |
| produced in oral spelling | | t-r (1) | o-a (3) | h-e (1) | c-i (1) |
| ($N = 32$) | | | o-e (1) | n-c (1) | d-g (1) |
| | | | | t-n (1) | e-r (1) |
| | | | | u-l (1) | i-e (2) |
| | | | | | l-o (1) |
| | | | | | n-i (1) |
| | | | | | o-y (1) |
| | | | | | q-c (2) |
| | | | | | r-c (1) |
| | | | | | r-l (2) |
| | | | | | r-u (1) |
| | | | | | s-a (1) |
| | | | | | s-c (1) |
| | | | | | t-a (1) |
| | | | | | t-l (1) |
| | | | | | u-i (1) |
